# Efficacy of Clotiazepam for Respiratory Dyskinesia in a Patient With Parkinsonism: A Case Report

**DOI:** 10.7759/cureus.103925

**Published:** 2026-02-19

**Authors:** Sekai Tsujimoto, Koji Hayashi, Yuka Nakaya, Asuka Suzuki, Mamiko Sato, Toyoaki Miura, Yasutaka Kobayashi

**Affiliations:** 1 Department of Rehabilitation Medicine, Fukui General Hospital, Fukui, JPN; 2 Graduate School of Health Sciences, Fukui Health Science University, Fukui, JPN

**Keywords:** benzodiazepine use, clotiazepam, #dyskinesia, parkinsonism, respiratory muscle

## Abstract

This report describes a rare case of respiratory dyskinesia in a patient with vascular parkinsonism that was successfully controlled with clotiazepam. An 89-year-old man with a history of vascular parkinsonism presented with dyspnea and irregular breathing associated with marked orofacial and truncal dyskinesia. These symptoms became prominent following the upward titration of a ropinirole patch to 24 mg/day. Although mirtazapine was subsequently added to address comorbid depressive symptoms, it failed to alleviate his respiratory distress. In contrast, the administration of clotiazepam (10 mg/day) resulted in rapid and marked improvement in both respiratory and peripheral dyskinetic movements within two days.

Respiratory dyskinesia is a rare motor complication of Parkinson’s disease and Parkinsonism that severely impairs quality of life. Despite its clinical impact, no established standard therapy exists, and responses to conventional treatments, including dopaminergic adjustments and other pharmacological approaches, remain variable. In particular, the clinical efficacy of management with clotiazepam, a short-acting thienodiazepine, has not been previously documented in the literature. Our findings suggest that the anxiolytic and GABA-mediated muscle-relaxant properties of clotiazepam, combined with its favorable safety profile for the elderly due to its short half-life, may provide a useful and effective new therapeutic option for the management of refractory respiratory dyskinesia. Although the limitations of drawing conclusions from a single case must be acknowledged, these results warrant further large-scale prospective studies to validate the efficacy and safety of clotiazepam in this population.

## Introduction

Parkinson’s disease and Parkinsonism are known to be associated with motor complications as treatment with L-DOPA is prolonged [1,2]. Dyskinesias affecting the limbs and trunk are among the representative motor complications and can substantially impair quality of life (QOL) [3]. A rarer but more distressing variant is respiratory dyskinesia (RD), caused by involuntary contractions of the respiratory muscles [4-6]. Characterized by irregular breathing, dyspnea, and a sense of "air hunger," RD often mimics primary cardiopulmonary disorders, posing a significant diagnostic challenge [6-10].

There is no established therapy for RD; adjustments of dopaminergic agents and symptomatic treatments such as amantadine or clonazepam have been attempted, but responses are variable [1-3,5]. In particular, drug sensitivity often changes in elderly patients and in those with vascular Parkinsonism, necessitating more cautious and strategic therapeutic choices compared to younger patients with idiopathic Parkinson’s disease [2,11]. Specifically, long-acting agents like clonazepam pose heightened risks of over-sedation and falls, limiting their utility in this vulnerable population [12].

Clotiazepam is a short-acting thienodiazepine that provides potent GABA_A_-mediated anxiolytic and muscle-relaxant effects [13]. Despite its favorable pharmacokinetic profile for elderly populations [13], its specific efficacy for RD has not been documented. This report describes an 89-year-old man with vascular parkinsonism whose refractory RD responded rapidly to clotiazepam, suggesting it may serve as a targeted, safer alternative to conventional long-acting benzodiazepines for managing RD in the elderly.

## Case presentation

An 89-year-old man who had a history of hypertension since age 70 and vascular parkinsonism since age 83 presented with dyspnea, oral discomfort, decreased appetite, and irritability and visited our hospital. He initially responded modestly to levodopa therapy, which improved his motor symptoms, but its efficacy had significantly declined in recent years. Eleven months prior to admission, a ropinirole patch was initiated at 8 mg/day and gradually titrated to 24 mg/day over the following two months. Concurrent with this upward titration, his orofacial and truncal dyskinetic movements became markedly prominent, accompanied by sialorrhea and distressing respiratory symptoms. To address his worsening motor symptoms and comorbid depression, additional antiparkinsonian medications and mirtazapine were introduced; however, mirtazapine failed to provide significant clinical improvement in either his mood or his respiratory distress.

**Figure 1:**
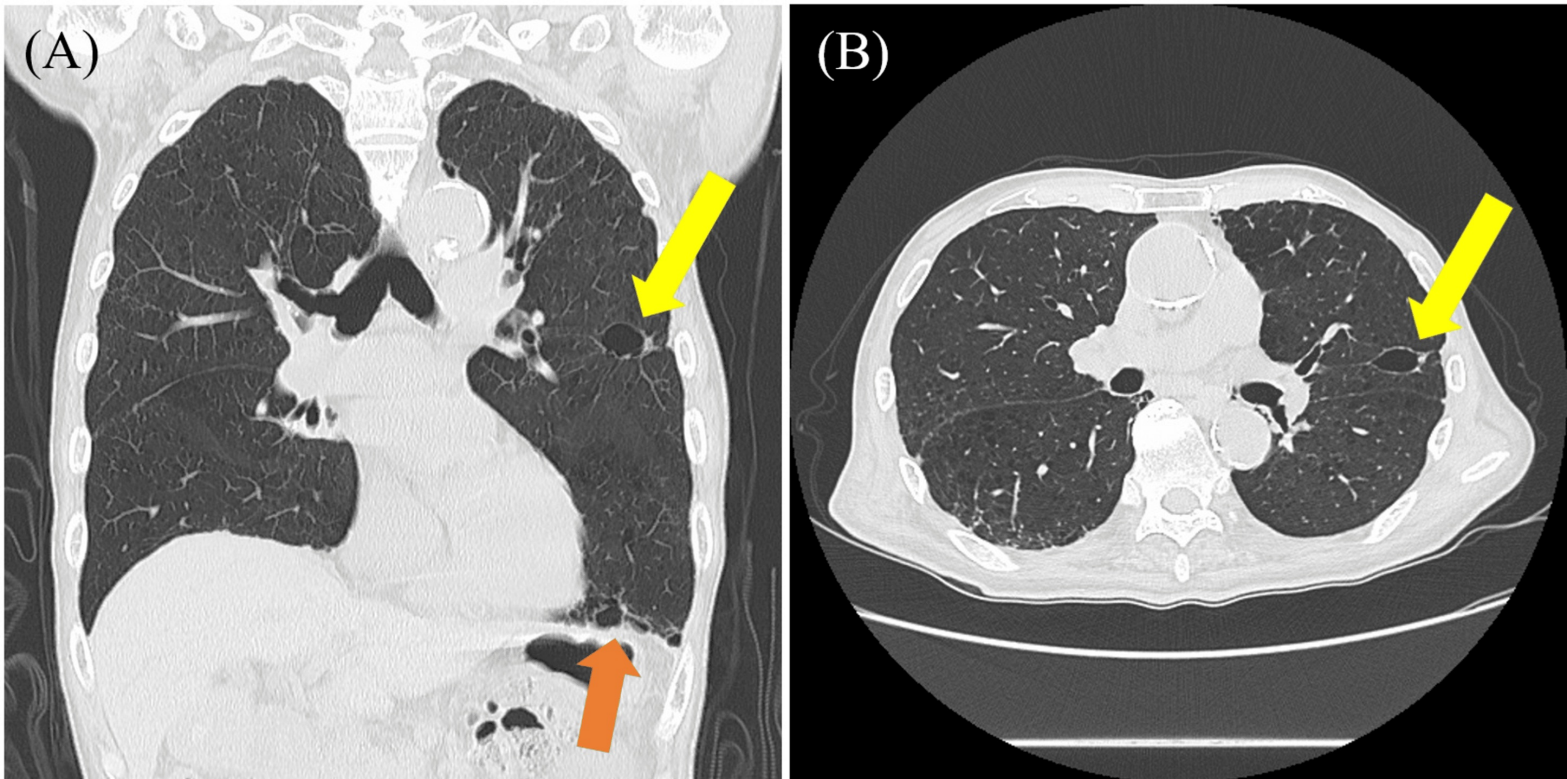
Thoracic computed tomography (CT) scan on lung window. (A, B) Chest CT scan showing pulmonary emphysematous changes (arrows). A: coronal section; B: axial section.

On admission, vital signs were as follows: body temperature 37.4°C, blood pressure 198/88 mmHg, pulse 81 bpm (regular rhythm), and SpO₂ 100% on room air. He demonstrated marked orofacial and truncal dyskinesia. Breathing was irregular, and he reported discomfort corresponding to the appearance of lip dyskinesia and truncal dyskinesia, but SpO₂ did not decrease. COVID-19 antigen and influenza antigen tests were negative. Laboratory results were within normal limits, including white blood cell count (6,500/μL; reference range 3,300-8,600/μL), C-reactive protein (0.06 mg/dL; 0.00-0.14 mg/dL), and liver function tests: aspartate aminotransferase (AST) 21 U/L (13-30 U/L) and alanine aminotransferase (ALT) 9 U/L (10-30 U/L). Chest computed tomography (CT) revealed pulmonary emphysematous changes, but no findings suggested active pneumonia (Figure 1). Based on these findings, we attributed his respiratory distress to dyskinesia, diagnosed as RD. In addition to respiratory symptoms, he experienced anxiety and was treated with clotiazepam 10 mg, a benzodiazepine medication commonly used in Japan. After two days of oral therapy, his anxiety was reduced, the dyspnea resolved, and there was a marked improvement in oral and truncal dyskinesia. The clinical course was favorable, and the patient was discharged on day 16. At a follow-up visit one month after discharge, the therapeutic effect of clotiazepam was maintained without recurrence of respiratory symptoms or adverse events such as over-sedation or falls.

Ethics statement: This study was conducted in accordance with the Declaration of Helsinki. Written informed consent was obtained from the patient for the publication of this case report, including clinical data and imaging findings. All identifying information has been removed or anonymized to ensure the protection of the patient’s identity.

## Discussion

This case describes a rare presentation of RD-a treatment-resistant involuntary movement of the respiratory musculature-in a patient with vascular parkinsonism, in whom rapid symptomatic control was achieved with oral clotiazepam. RD is an under-recognized variant of tardive dyskinesia (TD), characterized by involuntary contractions of the diaphragm and chest wall muscles [7]. It leads to irregular respiration, dyspnea, and "air hunger," symptoms that often disappear during sleep and are exacerbated by anxiety [5,9]. Although symptoms can be mild, RD is a potentially life-threatening condition [5].

The pathophysiology of RD involves postsynaptic dopamine D2 receptor hypersensitivity and up-regulation in the striatum, following long-term exposure to dopamine-receptor blocking agents or dopaminergic medications in Parkinson's disease [4,5]. While benzodiazepines like clonazepam are recommended for TD, their clinical utility in the extremely elderly is often limited by a long half-life (19-60 hours), which increases the risk of over-sedation, cognitive impairment, and falls [12].

Although the RD's phenomenon likely shares mechanisms with limb and truncal dyskinesias, no specific treatment targeting respiratory musculature has been firmly established [5,6,9]. Current RD treatments include discontinuation or reduction of causative neuroleptic medication, although this may temporarily worsen symptoms [4,5]. Pharmacological options involve tetrabenazine and benzodiazepines such as clonazepam, which reduce symptoms but are not curative [5,6,9]. In acute settings, haloperidol or intravenous diazepam may be used [2,5]. If antipsychotics must be continued, clozapine is an option due to its low TD risk [1,2]. For PD-related RD, successful management has been achieved using bilateral subthalamic nucleus (STN) deep brain stimulation (DBS) or unilateral globus pallidus internus (GPi) DBS [1,2]. Notably, while often used for Parkinsonian tremors, anticholinergics are known to exacerbate dyskinetic movements in TD [14], making them unlikely treatment options for RD.

A critical aspect of our therapeutic strategy was the selection of clotiazepam over other short-acting agents. Although midazolam is also a short-acting benzodiazepine, it is primarily utilized for acute sedation or in intravenous settings and lacks the clinical profile suitable for long-term oral maintenance therapy in a patient with chronic motor complications. In contrast, clotiazepam is a thienodiazepine that exerts potent GABAA-mediated muscle-relaxant and anxiolytic effects through the oral route [3]. Additionally, the clinical duration of clotiazepam (6-18 hours) remains substantially shorter than that of clonazepam (19-60 hours) [12,13,15]. This shorter profile might allow for more precise dose titration and reduce the risk of drug accumulation and toxicity in elderly patients with altered drug sensitivity. Furthermore, the patient’s normal liver function (AST 21 U/L, ALT 9 U/L) on admission might have ensured that the metabolism of this thienodiazepine could be managed safely, which is a prerequisite for initiating such therapy in the elderly.

Despite the dramatic clinical response observed within 48 hours, we must acknowledge the limitations of single-case inference. The efficacy of clotiazepam in this instance might be specifically linked to this patient’s unique combination of vascular parkinsonism and anxiety, which could have lowered his threshold for RD. As such, these findings should be interpreted with caution. Prospective studies or larger case series are urgently needed to validate the long-term efficacy and safety of clotiazepam and to determine its potential as a standardized therapeutic option for refractory RD in the broader Parkinsonian population.

## Conclusions

We report a rare case of an extremely elderly man with vascular Parkinsonism who developed refractory respiratory dyskinesia and showed marked improvement following the administration of clotiazepam, a benzodiazepine anxiolytic. While benzodiazepines, particularly clonazepam, have been utilized for the treatment of tardive dyskinesia, the efficacy of clotiazepam for respiratory dyskinesia has not been previously reported. Our findings suggest that the anxiolytic and GABA-mediated muscle-relaxant properties of clotiazepam may represent a valuable new therapeutic option for the management of refractory respiratory dyskinesia. However, it is important to acknowledge the limitations of drawing definitive conclusions from a single case report. Further large-scale prospective studies are required to validate the efficacy and long-term safety of clotiazepam and to establish it as a standardized treatment for this challenging condition.
